# Case Report: First report of *Legionella micdadei* pneumonia and organizing pneumonia in a patient with myelodysplastic and Sweet syndromes

**DOI:** 10.3389/fimmu.2025.1510948

**Published:** 2025-02-20

**Authors:** Yingying Chen, Sicong Liang, Ye Lu, Xiangyu Zhou, Rui Zheng, Yu Chen

**Affiliations:** ^1^ Department of Pulmonary and Critical Care Medicine, Shengjing Hospital of China Medical University, Shenyang, China; ^2^ Department of Critical Care Medicine, Shengjing Hospital of China Medical University, Shenyang, China; ^3^ Department of General Surgery, The Fourth Affiliated Hospital, China Medical University, Shenyang, Liaoning, China

**Keywords:** *Legionella pneumonia*, *Legionella micdadei*, organizing pneumonia, immunocompromised, Sweet syndrome

## Abstract

The immunocompromised population is susceptible to *Legionella* pneumonia. The diagnosis and treatment of *Legionella* pneumonia in immunocompromised individuals are challenging clinical endeavors. Previous studies have identified *Legionella* pneumonia as a potential cause of organizing pneumonia (OP), however, the association between *Legionella* pneumonia and OP has not received enough clinical attention. We retrospectively evaluated a case involving *Legionella micdadei* infection and OP in a patient with myelodysplastic syndrome and concurrent Sweet syndrome. The diagnosis of *Legionella micdadei* pneumonia was confirmed through various methods: metagenomic next generation sequencing (mNGS), Giemsa-staining and fluorescence *in situ* hybridization of lung tissue, as well as serum immunofluorescence antibody testing. Histopathological analysis of lung tissue revealed OP. The patient was successfully treated with a combination of antibiotics and low-dose glucocorticoids. In immunocompromised individuals, mNGS was capable of detection non-*Legionella* pneumophila serogroup 1. The pathological examination is important for identifying secondary OP and provides the evidence for treatment with glucocorticoids.

## Introduction

Immunocompromised hosts are susceptible to *Legionella* pneumonia, which has been widely described (1). The mortality rate of *Legionella* pneumonia in immunocompromised hosts is up to 50%, significantly higher than the 5–33% rate observed in the general population (2). However, due to the limitations of available detection methods, the diagnosis of *Legionella* infection, especially non-*Legionella pneumophila* (Lp) serogroup 1(Lp1), in immunocompromised hosts is challenging. *Legionella* infection can cause pulmonary interstitial changes, which are thought to be among the factors that lead to death (3). Although organizing pneumonia (OP) secondary to *Legionella* pneumonia has been reported (4), a comprehensive understanding of the clinical implications of this association is still lacking. Furthermore, the effect of modifying treatment strategies for secondary OP on the prognosis of *Legionella* pneumonia remains unclear.

We herein report a rare case of a *Legionella* pneumonia patient who was suffering from myelodysplastic syndrome (MDS) accompanied by Sweet syndrome. The diagnosis of *Legionella micdadei* pneumonia was established using multiple methods, including metagenomic next-generation sequencing(mNGS). The aim of this report is to enhance the comprehension of the relationship between *Legionella* pneumonia and OP. Furthermore, the report provides additional insights into the management and treatment strategies for patients with *Legionella* pneumonia who exhibit suboptimal responses to antibiotic therapy.

## Case description

**Figure 1:**
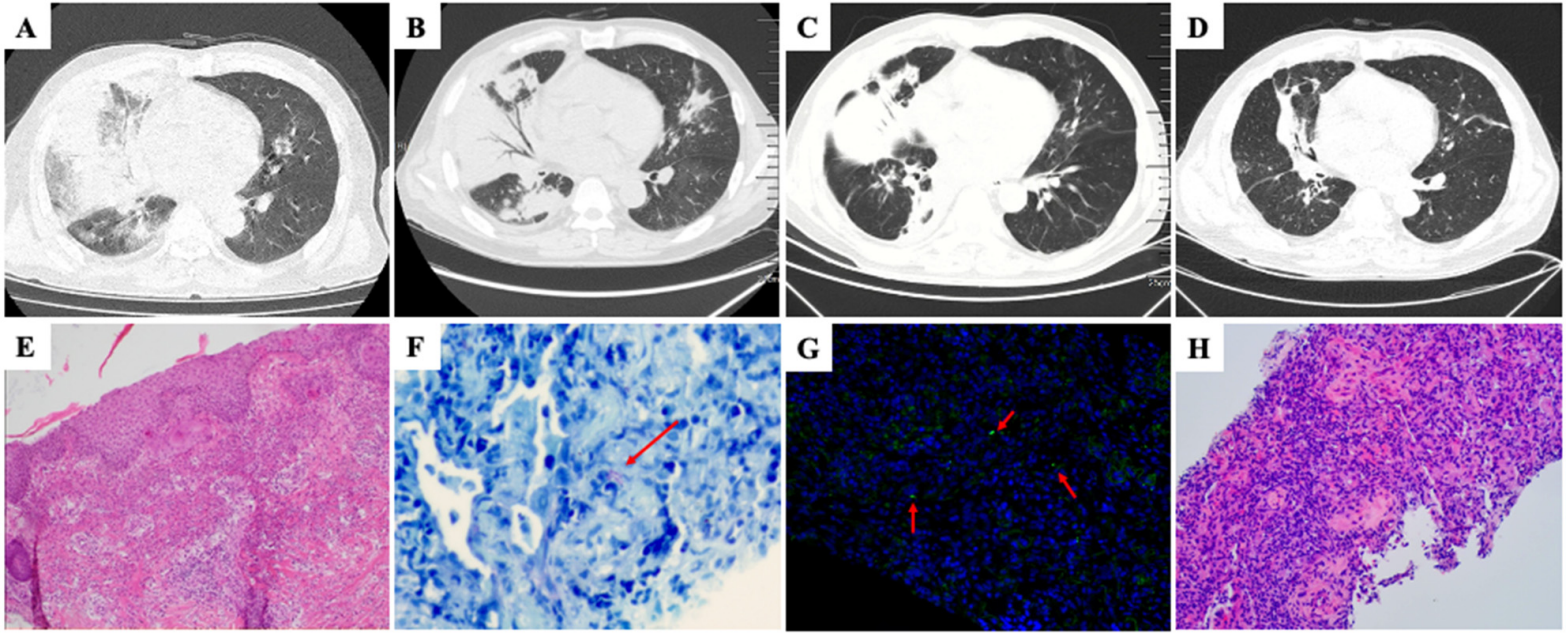
**(A)** Chest CT scan (March 30, 2021) showed multiple ground glass opacities and consolidation in right middle and lower lobes. **(B)** Post-admission chest CT scan, following a two-week course of antibiotic treatment, revealed a reduction in ground-glass opacities and an increase in consolidation. **(C)** Chest CT performed after five-day intensive antibiotics treatment for *Legionella* pneumonia showed decreased consolidation and reduced right lung volume. **(D)** Follow-up chest CT performed after seven weeks of glucocorticoid treatment showed markedly decreased extent of abnormal opacity in right lung. **(E)** Histopathological analysis of skin biopsy showed dense dermal infiltration of neutrophils without overt vasculitis (hematoxylin and eosin stain, magnification × 100). **(F)** Giemsa staining of lung tissue showed presence of intracellular slender bacilli (arrow) suggestive of *Legionella* (magnification × 400). **(G)** Specific identification of *Legionella micdadei* (arrow) in lung biopsy tissue by fluorescence *in situ* hybridization (magnification × 400). **(H)** Lung biopsy showed buds of granulation tissue occupying alveolar spaces (hematoxylin and eosin stain, magnification × 200).

A 58-year-old male Chinese sanitation worker initially presented to our fever clinic on March 30, 2021, reporting recurrent fever lasting for over a year. The fever typically occurred in the afternoon, with a daily maximum temperature between 37.5 and 37.6°C, without respiratory system symptoms or other accompanying symptoms. He was referred to a local hospital for medical evaluation and diagnosed with anemia. The patient was intermittently administered cephalosporins, traditional Chinese medicine, and dexamethasone. Despite achieving a normal temperature for approximately one week, his condition relapsed. The patient was committed to environmental sanitation duties. On March 24, 2021, the patient’s temperature reached a record high of 39.5°C and was accompanied by bloody sputum. Consequently, he sought medical attention at our fever clinic, where routine blood tests revealed anemia with a hemoglobin level of 75 g/L and thrombocytopenia with a platelet count of 59*10^9/L. Bone marrow aspiration revealed no abnormal primary cells, and the ratio of granulocytes to erythrocytes increased significantly. The next-generation sequencing of the marrow showed a gene mutation in the DNMT3A gene (with a 41.28% variant allele frequency). Therefore, the patient was diagnosed as having MDS. Additionally, chest CT revealed consolidation and ground-glass opacity in the right lung (**Figure 1A**) and elevated C-reactive protein (272.0 mg/L) and procalcitonin (0.93ng/mL). Despite receiving a two-week regimen of intravenous cephalosporin and levofloxacin, the patient’s fever persisted. Subsequently, the patient was transferred to the respiratory department on April 14, 2021. The patient presented with no prior medical history and explicitly denied any history of pulmonary disease. He had not undergone any previous chest X-ray examinations. Additionally, he denied any history of pet ownership or substance abuse. There was no family history of pulmonary disease or cancer reported. The blood gas analysis revealed type I respiratory failure with a PaO_2_ of 63.9 mmHg while receiving 2 L/min nasal oxygen therapy (oxygenation index: 220.34 mmHg). Serum 1-3-beta-D-glucan and galactomannan tests and interferon-gamma release assays yielded negative results. Sputum culture results were negative. The primary laboratory results are listed in **Table 1**.

**Table 1:** The main laboratory results of the patient on admission.

Items	Value	Reference range	Items	Value	Reference range
Blood routine			Blood biochemical test		
WBC counts (10^9^/L)	7.6	3.5-9.5	Potassium (mmol/L)	3.47	3.5 - 5.5
Neutrophil (%)	72.8	43.2 – 71.5	Sodium (mmol/L)	135.1	136 - 145
Hemoglobin (g/L)	68	130-172	Urea(mmol/L)	2.77	3 - 9.2
Platelet(10^9^/L)	43	135 - 350	Creatinine (μmol/L)	50	59 - 104
C-reactive protein(mg/L)	130.8	0-8	Total protein (g/L)	65.3	60 - 83
Procalcitonin	0.510	<0.05	ALT (U/L)	24	0 - 40
ESR (mm/H)	100	0 - 15	AST (U/L)	29	5 - 34
D-dimer (μg/L)	1205	0 - 252	Albumin (g/L)	27.7	35 - 53
Arterial blood gas analysis			LDH	392	80-285
PH	7.456	7.35 – 7.45	Serum HIV antibody	Negative	Negative
PaO_2_ (mmHg)	63.9	80 - 100	T-spot	Positive	Negative
PaCO_2_ (mmHg)	38.9	35 - 45	Panel A of ESAT6	6	0-6
Serum anti-*Mycoplasma* IgM	Negative	Negative	Panel B of CFP-10	0	0-6
Serum anti-*Chlamydia* IgM	Negative	Negative	Serum 1-3-β- glucan (pg/mL)	<37.5	<70
Serum anti-*Legionella* IgG	Negative	Negative	Serum galactomannan (ug/L)	≤0.25	<0.65
Sputum culture of bacterium	Negative	Negative	Immunological parameters		
Sputum examination	Negative	Negative	ANA titer	Negative	Negative
Sputum smear of TB	Negative	Negative	ANA spectrum	Negative	Negative
Sputum culture of fungus	Negative	Negative	ANCA	Negative	Negative
Urine antigen for *Legionella*	Negative	Negative	Blood culture	Negative	Negative

WBC, white blood cell; ESR, Erythrocyte sedimentation rate; ALT, Alanine aminotransferase; AST, Aspartate aminotransferase; LDH, lactate dehydrogenase; IgM, Immunoglobulin M; IgG, Immunoglobulin G; TB, tuberculosis; HIV, human immunodeficiency virus; PaO_2_, arterial partial pressure of oxygen; PaCO_2_, arterial Partial Pressure of Carbon Dioxide; ANCA, Anti-neutrophil cytoplasmic antibody; ANA, Antinuclear antibody.

The patient presented with flat papules of dark purplish-red coloration distributed on both upper limbs, neck, front chest, back shoulder, and buttocks. Histopathological examination of the skin revealed Sweet syndrome (**Figure 1E**).

The patient was treated with ubenimex and stanozolol for MDS. Nevertheless, following a two-week course of antibiotic therapy, the patient’s body temperature persisted, and a subsequent review of CT scans revealed a reduction in ground-glass opacities and an increase in consolidation (**Figure 1B**). Subsequently, a percutaneous aspiration lung biopsy was performed. mNGS of lung tissue detected *Legionella micdadei.* Based on the literature (5–7), the fluorescence *in situ* hybridization (FISH) probe for *Legionella micdadei* was prepared as follows: FAM-AGCTGATTGGTTAATAGCCCAATCGG-FAM. Giemsa staining (**Figure 1F**) and FISH-probe of lung tissue (**Figure 1G**) confirmed *Legionella micdadei* infection. In addition, the patient’s serum immunofluorescent antibody to *Legionella micdadei* was positive, whereas the *Legionella* urinary antigen was negative. *Legionella micdadei* pneumonia was confirmed. The patient received a regimen of moxifloxacin and tigecycline for the management of *Legionella* pneumonia.

Despite undergoing five-day intensive antibiotic treatment for *Legionella* pneumonia, the patient experienced recurrent episodes of high fever. Repeated full blood count testing a white cell count was 8.6× 10^9^/L with a neutrophil ratio of 76.4%, and the CRP level decreased to 70.4mg/L. Additionally, chest CT showed that the consolidation had decreased alongside a reduction in the right lung volume, suggesting the possibility of OP (**Figure 1C**). Subsequently, pathological examination of the patient’s lung tissue revealed OP changes (**Figure 1H**). Therefore, the patient was administered a ten-day course of intravenous methylprednisolone at a daily dose of 40 mg in conjunction with antibiotics. The patient’s temperature normalized on the first day of methylprednisolone treatment. Consequently, the intravenous methylprednisolone was replaced with oral prednisone tablets (40 mg daily). The patient was discharged without complications. Subsequently, the prednisone dosage was gradually decreased by 5 mg every two weeks post-discharge, with no reported adverse effects observed during the treatment period. A chest CT scan performed after seven weeks of glucocorticoid treatment revealed notable resolution of the pulmonary radiographic findings (**Figure 1D**).

## Discussion

To the best of our knowledge, this is the first report of a patient with MDS and Sweet syndrome diagnosed with *Legionella micdadei* pneumonia and OP.

The diagnosis of *Legionella* pneumonia frequently encounters obstacles stemming from both underdiagnosis and delayed diagnosis, primarily attributable to the absence of easily accessible diagnostic tools for the early detection of all *Legionella* serogroups and species capable of causing infections. In addition to distinct clinical manifestations, a detailed environmental exposure history is a crucial factor in the diagnostic process and warrants careful attention. The patient had been engaged in sanitation duties and had a history of environmental exposure to sewage, which may have been the origin of his infection with *Legionella micdadei*. The *Legionella micdadei* is the second most common species associated with *Legionella* pneumonia (8). This species has been found to be associated with more immunocompromised patients and was not identified by the first-line screening test, *Legionella* urinary antigen. Therefore, it is critical to use specific methods to identify various *Legionella* species, especially in immunocompromised individuals. Recently, mNGS has emerged as a valuable tool for the identification of difficult-to-culture pathogens. Through the use of advanced nucleic acid detection and molecular techniques, mNGS offers heightened sensitivity, specificity, and expedited pathogen detection capacity (9). Advances in pathogen identification technology will reveal the etiological pathogens of pneumonia in immunocompromised populations.

OP is characterized by a pattern of lung tissue repair, which often occurs as a result of a specific injury (such as infection, connective tissue disorder, aspiration, and drug toxicity) or from an unknown source (cryptogenic) (10). The predominant etiology of secondary OP is an infection. *Legionella* includes a host of pathogens that can cause OP (10). Previous studies of *Legionella* pneumonia have shown that its pathological alterations include diffuse alveolar damage, fibrin deposition, and fibrosis in severe cases (3, 11). Pulmonary interstitial changes due to *Legionella* pneumonia have been recognized for some time and can lead to disease progression and potentially fatal outcomes (3). However, *Legionella* pneumonia-associated pulmonary interstitial changes have not received sufficient clinical attention.

Moreover, a previous study described the occurrence of OP in patients with MDS accompanied by Sweet syndrome (12). Sweet Syndrome, also known as “acute febrile neutrophilic dermatosis,” is often linked to malignant hematological conditions such as MDS, likely driven by autoimmunity and immunodeficiency (13). Interestingly, Sweet syndrome and OP can precede, coincide, or follow each other during MDS progression (12). The mechanisms underlying these conditions require further investigation.

The causes of OP formation in this patient were complex and likely related to both *Legionella* pneumonia and underlying MDS and Sweet syndrome. The initial chest CT results revealed a relatively sharp-bordered consolidation intermixed with ground-glass opacity, consistent with the chest imaging characteristics of acute *Legionella* pneumonia (14). Therefore, we speculated that *Legionella* infection was a significant factor in the development of OP in this patient, with MDS and Sweet syndrome potentially exerting a secondary facilitating effect.

The patient experienced a sustained fever despite treatment with a combination of antibiotics. Subsequent pathological findings indicated a diagnosis of OP, with symptoms and imaging showing improvement following glucocorticoid therapy. Therefore, in cases of *Legionella* pneumonia patients where clinical manifestations persist despite appropriate anti-*Legionella* treatment and imaging indicates the possibility of OP, active histopathological examination is essential to adjust the treatment regimen and improve the prognosis.

## Conclusion

The number of immunocompromised individuals is steadily increasing, highlighting the increasing importance of recognizing the latent risk of *Legionella* pneumonia. mNGS has important application value in the detection of rare pathogens of pneumonia in immunocompromised individuals. For patients with *Legionella* pneumonia who do not respond well to a combination of antibiotic treatments, pathological analysis can aid in identifying potential secondary OP and provide evidence for treatment with glucocorticoid.

## Data Availability

The raw data supporting the conclusions of this article will be made available by the authors, without undue reservation.
